# Supplementary material for: Utility of the SARC-F Questionnaire for Sarcopenia Screening in Patients with Chronic Liver Disease: A Multicenter Cross-Sectional Study in Japan
Source: J Clin Med. 2021 Aug 3;10(15):3448. doi: 10.3390/jcm10153448 (PMC8348368; doi:10.3390/jcm10153448)
Supplement: Supplementary file 1 [file jcm-10-03448-s001.zip › jcm-1291838-supplementary.pdf]

Journal of Clinical Medicine

Table S1. Comparison of body compositions of patients with and without sarcopenia.

Characteristics	Patients	Total cohort	No sarcopenia	Sarcopenia	P–value*
Handgrip strength, kg	676	26.7 (20.0–34.3)	29.1 (21.5–35.2)	17.8 (15.1–22.8)	<0.001
Muscle mass					
Skeletal muscle index, cm²/m²	56 (8%)	43.3 (37.9–52.7)	44.3 (39.0–53.3)	32.0 (31.4–36.0)	<0.001
Psoas muscle index, cm²/m²	383 (57%)	4.9 (3.8–5.8)	5.1 (4.0–6.1)	3.9 (3.3–5.0)	<0.001
Appendicular skeletal muscle index, kg²/m²	237 (35%)	8.6 (7.3–9.9)	8.8 (7.4–10.0)	5.6 (5.3–6.3)	<0.001

Values are presented as number (percentage) or median (interquartile range). *Mann–Whitney U test was used to compare clinical characteristics between the two groups.

Table S2. Comparison of body compositions of patients with SARC-F < 4 and those with ≥ 4.

Characteristics	Patients	Total Cohort	SARC-F < 4	SARC-F ≥ 4	P–value*
Handgrip strength, kg	676	26.7 (20.0–34.3)	27.8 (21.1–34.7)	15.6 (12.7–20.9)	<0.001
Muscle mass					
Skeletal muscle index, cm²/m²	56 (8%)	43.3 (37.9–52.7)	44.0 (38.2–52.7)	41.8 (36.5–50.7)	0.675
Psoas muscle index, cm²/m²	383 (57%)	4.9 (3.8–5.8)	8.7 (7.3–10.0)	7.6 (6.5–8.5)	0.003
Appendicular skeletal muscle index, kg²/m²	237 (35%)	8.6 (7.3–9.9)	4.9 (3.9–5.8)	3.8 (3.3–5.2)	0.007

Values are presented as number (percentage) or median (interquartile range). *Mann–Whitney U test was used to compare clinical characteristics between the two groups.

Table S3. Baseline characteristics of patients with and without sarcopenia according to sex.

	Men (N = 458)			Women (N = 218)		
	No sarcopenia (N = 399)	Sarcopenia (N = 59)	P–value*	No sarcopenia (N = 176)	Sarcopenia (N = 42)	P–value*
Characteristics						
Age, years	69 (61–77)	78 (73–85)	< 0.001	71 (64–78)	79 (74–82)	< 0.001
Body mass index, kg/m²	23.7 (21.8–26.3)	22.0 (20.2–23.7)	< 0.001	23.7 (21.1–26.7)	21.4 (19.3–24.1)	< 0.001
Etiology			0.012			0.172
Hepatitis B virus	75 (19)	2 (3)		17 (10)	5 (12)	
Hepatitis C virus	111 (28)	20 (34)		60 (34)	21 (50)	
Alcohol-related	82 (21)	13 (22)		14 (8)	1 (2)	
Others	131 (33)	24 (41)		85 (48)	15 (36)	
Liver cirrhosis	231 (58)	44 (75)	0.015	116 (66)	30 (71)	0.585
Child-Pugh class†			0.428			0.485
A	338 (85)	47 (80)		141 (80)	31 (74)	
B	50 (13)	11 (19)		28 (16)	8 (19)	
C	11 (3)	1 (2)		7 (4)	3 (7)	
Hepatocellular carcinoma	268 (67)	45 (76)	0.179	84 (48)	28 (67)	0.038
SARC-F components						
Strength			0.001			0.005
None (0)	366 (92)	45 (76)		142 (81)	24 (57)	
Some (1)	23 (6)	13 (22)		26 (15)	15 (36)	
A lot or unable (2)	10 (3)	1 (2)		8 (5)	3 (7)	
Assistance in walking			0.001			0.016
None (0)	378 (95)	47 (80)		156 (89)	30 (71)	

Some (1)	18 (5)	11 (19)		18 (10)	11 (26)	
A lot, use aids, or unable (2)	3 (1)	1 (2)		2 (1)	1 (2)	
Rise from a chair			0.003			0.001
None (0)	369 (93)	46 (78)		153 (87)	27 (64)	
Some (1)	28 (7)	12 (20)		23 (13)	15 (36)	
A lot or unable without help (2)	2 (1)	1 (2)		0 (0)	0 (0)	
Climb stairs			0.002			0.009
None (0)	336 (84)	38 (64)		132 (75)	23 (55)	
Some (1)	52 (13)	18 (31)		41 (23)	15 (36)	
A lot or unable (2)	11 (3)	3 (5)		3 (2)	4 (10)	
Falls			< 0.001			0.141
None (0)	350 (88)	37 (63)		137 (78)	27 (64)	
1–3 falls (1)	47 (12)	22 (37)		37 (21)	14 (33)	
4 or more falls (2)	2 (1)	0 (0)		2 (1)	1 (2)	

Values are presented as number (percentage) or median (interquartile range). †Patients with cirrhosis were evaluated. *The chi-square test for categorical variables or Mann–Whitney U test for continuous variables was used to compare clinical characteristics between the two groups.

Table S4. Sensitivity, specificity, and positive and negative predictive values for sarcopenia according to sex.

	Men (N = 458)			
	Sensitivity (95% CI)	Specificity (95% CI)	PPV (95% CI)	NPV (95% CI)
SARC-F ≥ 1	0.58 (0.44–0.70)	0.74 (0.69–0.78)	0.25 (0.18–0.33)	0.92 (0.89–0.95)
SARC-F ≥ 2	0.34 (0.22–0.47)	0.88 (0.84–0.91)	0.29 (0.19–0.41)	0.90 (0.87–0.93)
SARC-F ≥ 3	0.22 (0.12–0.35)	0.93 (0.89–0.95)	0.30 (0.17–0.46)	0.89 (0.86–0.92)
SARC-F ≥ 4	0.15 (0.07–0.27)	0.96 (0.94–0.98)	0.36 (0.18–0.58)	0.89 (0.85–0.91)
	Women (N = 218)			
	Sensitivity (95% CI)	Specificity (95% CI)	PPV (95% CI)	NPV (95% CI)
SARC-F ≥ 1	0.76 (0.61–0.88)	0.56 (0.48–0.63)	0.29 (0.21–0.39)	0.91 (0.84–0.96)
SARC-F ≥ 2	0.45 (0.30–0.61)	0.76 (0.69–0.82)	0.31 (0.20–0.44)	0.85 (0.79–0.90)
SARC-F ≥ 3	0.36 (0.22–0.52)	0.86 (0.80–0.91)	0.39 (0.23–0.55)	0.85 (0.79–0.90)
SARC-F ≥ 4	0.29 (0.16–0.45)	0.90 (0.84–0.94)	0.40 (0.23–0.59)	0.84 (0.78–0.89)

CI, confidence interval; NPV, negative predictive value; PPV, positive predictive value.

Table S5. Baseline characteristics of patients with and without sarcopenia according to age.

	Age < 65 years (N = 183)			Age ≥ 65 years (N = 493)		
	No sarcopenia (N = 174)	Sarcopenia (N = 9)	P–value*	No sarcopenia (N = 401)	Sarcopenia (N = 92)	P–value*
Characteristics						
Age, years	56 (50–61)	61 (61–63)	0.022	74 (70–79)	79 (75–84)	<0.001
Body mass index, kg/m²	24.1 (21.6–27.1)	21.2 (19.3–22.4)	0.005	23.6 (21.7–26.3)	21.8 (19.8–24.0)	<0.001
Male sex	129 (74)	5 (56)	0.252	270 (67)	54 (59)	0.144
Etiology			0.408			0.184
Hepatitis B virus	33 (19)	0 (0)		59 (15)	7 (8)	
Hepatitis C virus	38 (22)	3 (33)		133 (33)	38 (41)	
Alcohol-related	35 (20)	3 (33)		61 (15)	11 (12)	
Others	68 (39)	3 (33)		148 (37)	36 (39)	

Liver cirrhosis	92 (53)	8 (89)	0.042	255 (64)	66 (72)	0.147
Child-Pugh class†			0.005			0.301
A	139 (80)	3 (33)		340 (85)	75 (82)	
B	22 (13)	5 (56)		56 (14)	14 (15)	
C	13 (8)	1 (11)		5 (1)	3 (3)	
Hepatocellular carcinoma	69 (40)	5 (56)	0.488	283 (71)	68 (74)	0.610
SARC-F components						
Strength			< 0.001			0.001
None (0)	165 (95)	4 (44)		343 (86)	65 (71)	
Some (1)	7 (4)	5 (56)		42 (11)	23 (25)	
A lot or unable (2)	2 (1)	0 (0)		16 (4)	4 (4)	
Assistance in walking			< 0.001			0.002
None (0)	169 (97)	5 (56)		365 (91)	72 (78)	
Some (1)	5 (3)	4 (44)		31 (8)	18 (20)	
A lot, use aids, or unable (2)	0 (0)	0 (0)		5 (1)	2 (2)	
Rising from a chair			< 0.001			0.004
None (0)	171 (98)	5 (56)		351 (88)	68 (74)	
Some (1)	3 (2)	4 (44)		48 (12)	23 (25)	
A lot or unable without help (2)	0 (0)	0 (0)		2 (1)	1 (1)	
Climbing stairs			0.003			0.007
None (0)	163 (94)	5 (56)		305 (76)	56 (61)	
Some (1)	10 (6)	4 (44)		83 (21)	29 (32)	
A lot or unable (2)	1 (1)	0 (0)		13 (3)	7 (8)	
Falls			0.011			0.001
None (0)	158 (91)	5 (56)		329 (82)	59 (64)	
1–3 falls (1)	14 (8)	4 (44)		70 (18)	32 (35)	
4 or more falls (2)	2 (1)	0 (0)		2 (1)	1 (1)	

Values are presented as number (percentage) or median (interquartile range). †Patients with cirrhosis were evaluated. *The chi-square test for categorical variables or Mann–Whitney U test for continuous variables were used to compare clinical characteristics between the two groups.

Table S6. Sensitivity, specificity, and positive and negative predictive values for sarcopenia according to age.

	Age < 65 years (N = 183)			
	Sensitivity (95% CI)	Specificity (95% CI)	PPV (95% CI)	NPV (95% CI)
SARC-F ≥ 1	0.67 (0.30–0.93)	0.85 (0.78–0.90)	0.18 (0.07–0.36)	0.98 (0.94–0.99)
SARC-F ≥ 2	0.44 (0.14–0.79)	0.95 (0.90–0.98)	0.31 (0.09–0.61)	0.97 (0.93–0.99)
SARC-F ≥ 3	0.44 (0.14–0.79)	0.97 (0.93–0.99)	0.44 (0.14–0.79)	0.97 (0.93–0.99)
SARC-F ≥ 4	0.44 (0.14–0.79)	0.98 (0.95–0.99)	0.57 (0.18–0.90)	0.97 (0.94–0.99)
	Age ≥ 65 years (N = 493)			
	Sensitivity (95% CI)	Specificity (95% CI)	PPV (95% CI)	NPV (95% CI)
SARC-F ≥ 1	0.65 (0.55–0.75)	0.61 (0.56–0.66)	0.28 (0.22–0.34)	0.89 (0.84–0.92)
SARC-F ≥ 2	0.38 (0.28–0.49)	0.79 (0.75–0.83)	0.30 (0.22–0.39)	0.85 (0.81–0.88)
SARC-F ≥ 3	0.26 (0.18–0.36)	0.88 (0.84–0.91)	0.33 (0.22–0.45)	0.84 (0.80–0.87)
SARC-F ≥ 4	0.19 (0.11–0.28)	0.92 (0.89–0.95)	0.35 (0.22–0.51)	0.83 (0.79–0.87)

CI, confidence interval; NPV, negative predictive value; PPV, positive predictive value.

Table S7. Baseline characteristics of patients with and without sarcopenia according to sarcopenia diagnostic methods.

	Skeletal muscle index (N = 56)			Psoas muscle index (N = 383)			Appendicular skeletal muscle mass index (N = 237)		
	No sarcopenia (N = 49)	Sarcopenia (N = 7)	P–value*	No sarcopenia (N = 303)	Sarcopenia (N = 80)	P–value*	No sarcopenia (N = 223)	Sarcopenia (N = 14)	P–value*
Characteristics									
Age, years	74 (68–79)	79 (76–79)	0.180	70 (62–77)	78 (72–83)	<0.001	70 (60–77)	84 (77–86)	<0.001
Body mass index, kg/m²	24.2 (21.9–27.1)	22.8 (20.6–26.1)	0.407	23.4 (21.5–25.6)	22.0 (19.9–24.0)	<0.001	24.1 (21.9–27.3)	20.6 (18.9–21.8)	<0.001
Male sex	30 (61)	2 (29)	0.125	209 (69)	50 (63)	0.284	160 (72)	7 (50)	0.127
Etiology			0.192			0.003			0.341
Hepatitis B virus	15 (31)	1 (14)		46 (15)	4 (5)		31 (14)	2 (14)	
Hepatitis C virus	9 (18)	4 (57)		152 (50)	35 (44)		10 (5)	2 (14)	
Alcohol-related	8 (16)	0 (0)		50 (17)	13 (16)		38 (17)	1 (7)	
Others	17 (35)	2 (29)		55 (18)	28 (35)		144 (65)	9 (64)	
Liver cirrhosis	41 (84)	6 (86)	1.000	162 (54)	55 (69)	0.016	144 (65)	13 (93)	0.039
Child-Pugh class†			0.201			0.090			0.832
A	34 (69)	3 (43)		269 (89)	64 (80)		176 (79)	11 (79)	
B	13 (27)	3 (43)		26 (9)	13 (16)		39 (18)	3 (21)	
C	2 (4)	1 (14)		8 (3)	3 (4)		8 (4)	0 (0)	
Hepatocellular carcinoma	29 (59)	2 (29)	0.223	172 (57)	57 (71)	0.021	151 (68)	14 (100)	0.007
SARC-F components									
Strength			0.190			<0.001			0.033
None (0)	30 (61)	3 (43)		284 (94)	57 (71)		194 (87)	9 (64)	
Some (1)	12 (25)	1 (14)		19 (6)	23 (29)		18 (8)	4 (29)	
A lot or unable (2)	7 (14)	3 (43)		0 (0)	0 (0)		11 (5)	1 (7)	
Assistance in walking			0.696			<0.001			0.010
None (0)	39 (80)	5 (71)		289 (95)	62 (78)		206 (92)	10 (71)	
Some (1)	8 (16)	2 (29)		14 (5)	18 (23)		14 (6)	2 (14)	
A lot, use aids, or unable (2)	2 (4)	0 (0)		0 (0)	0 (0)		3 (1)	2 (14)	
Rising from a chair			0.658			<0.001			0.001
None (0)	38 (78)	5 (71)		279 (92)	60 (75)		205 (92)	8 (57)	
Some (1)	11 (22)	2 (29)		24 (8)	20 (25)		16 (7)	5 (36)	
A lot or unable without help (2)	0 (0)	0 (0)		0 (0)	0 (0)		2 (1)	1 (7)	
Climbing stairs			0.272			<0.001			<0.001
None (0)	28 (57)	3 (43)		254 (84)	52 (65)		186 (83)	6 (43)	
Some (1)	17 (35)	2 (29)		49 (16)	28 (35)		27 (12)	3 (21)	
A lot or unable (2)	4 (8)	2 (29)		0 (0)	0 (0)		10 (5)	5 (36)	
Falls			0.729			<0.001			0.001
None (0)	37 (76)	5 (71)		263 (87)	53 (66)		187 (84)	6 (43)	
1–3 falls (1)	10 (20)	2 (29)		40 (13)	27 (34)		34 (15)	7 (50)	
4 or more falls (2)	2 (4)	0 (0)		0 (0)	0 (0)		2 (1)	1 (7)	

Values are presented as number (percentage) or median (interquartile range). †Patients with cirrhosis were evaluated. *The chi-square test for categorical variables or Mann–Whitney U test for continuous variables were used to compare clinical characteristics between the two groups.

Table S8. Sensitivity, specificity, and positive and negative predictive values for sarcopenia according to sarcopenia diagnostic methods.

	Skeletal muscle index (N = 56)			
	Sensitivity (95% CI)	Specificity (95% CI)	PPV (95% CI)	NPV (95% CI)
SARC-F ≥ 1	0.71 (0.29–0.96)	0.71 (0.29–0.96)	0.15 (0.05–0.31)	0.91 (0.71–0.99)
SARC-F ≥ 2	0.57 (0.18–0.90)	0.57 (0.42–0.71)	0.16 (0.05–0.36)	0.90 (0.74–0.98)
SARC-F ≥ 3	0.43 (0.10–0.82)	0.74 (0.59–0.85)	0.19 (0.04–0.46)	0.90 (0.76–0.97)
SARC-F ≥ 4	0.43 (0.10–0.82)	0.82 (0.68–0.91)	0.25 (0.06–0.57)	0.91 (0.78–0.98)
	Psoas muscle index (N = 383)			
	Sensitivity (95% CI)	Specificity (95% CI)	PPV (95% CI)	NPV (95% CI)
SARC-F ≥ 1	0.64 (0.52–0.74)	0.74 (0.68–0.79)	0.39 (0.31–0.48)	0.89 (0.84–0.92)
SARC-F ≥ 2	0.34 (0.24–0.45)	0.87 (0.82–0.90)	0.40 (0.28–0.52)	0.83 (0.79–0.87)
SARC-F ≥ 3	0.23 (0.14–0.33)	0.95 (0.92–0.97)	0.53 (0.35–0.70)	0.82 (0.78–0.86)
SARC-F ≥ 4	0.16 (0.09–0.26)	0.97 (0.92–0.96)	0.59 (0.36–0.79)	0.81 (0.77–0.85)
	Appendicular skeletal muscle mass index (N = 237)			
	Sensitivity (95% CI)	Specificity (95% CI)	PPV (95% CI)	NPV (95% CI)
SARC-F ≥ 1	0.71 (0.42–0.92)	0.67 (0.61–0.73)	0.12 (0.06–0.21)	0.97 (0.94–0.99)
SARC-F ≥ 2	0.57 (0.29–0.82)	0.87 (0.81–0.91)	0.21 (0.10–0.37)	0.97 (0.94–0.99)
SARC-F ≥ 3	0.50 (0.23–0.77)	0.89 (0.84–0.93)	0.22 (0.09–0.40)	0.97 (0.93–0.99)
SARC-F ≥ 4	0.36 (0.13–0.65)	0.93 (0.89–0.96)	0.24 (0.08–0.47)	0.96 (0.92–0.98)

CI, confidence interval; NPV, negative predictive value; PPV, positive predictive value.
